# Avian species survey with citizen-science data in Janghang Wetland, Goyang, Republic of Korea

**DOI:** 10.3897/BDJ.11.e105580

**Published:** 2023-05-31

**Authors:** Hyun-Ah Choi, Eunjeong Lee, Eunjeong Kim, Insook Jung, Donguk Han

**Affiliations:** 1 Hanns Seidel Foundation Korea, Seoul, Republic of Korea Hanns Seidel Foundation Korea Seoul Republic of Korea; 2 OJEong Resilience Institute, Korea University, Seoul, Republic of Korea OJEong Resilience Institute, Korea University Seoul Republic of Korea; 3 PGA Eco and Bio Diversity Institute, ECO Korea, Goyang, Republic of Korea PGA Eco and Bio Diversity Institute, ECO Korea Goyang Republic of Korea; 4 Kongju National University, Gongju, Republic of Korea Kongju National University Gongju Republic of Korea

**Keywords:** Han River estuary, waterbirds, brackish water zone, conservation, citizen science

## Abstract

**Background:**

Monitoring of avian populations in Janghang Wetland, Goyang, Republic of Korea (ROK) is based on citizen science (also called community-based monitoring). This monitoring data can be used to track avian density, population status and waterbird census at local, national and regional levels. The Ministry of Environment (MoE) ROK has surveyed since 1999, including Odusan Unification Tower to Ilsan Bride, which connects the cities of Gimpo and Goyang along the Han River estuary. However, it has not covered Janghang Wetland, which is located in the Han River estuary at the transboundary between the two Koreas. The Janghang Wetland is a protected wetland in the Demilitarized Zone (DMZ) between the two Koreas. In 2019, Janghang Wetland was designated as a Flyway Network Site by Goyang City and the East Asian-Australasian Flyway Partnership. This Network site is a voluntary collaboration and includes many internationally significant wetlands for waterbirds that still lack formal national protection. In addition, it was designated as a Ramsar site in 2021. The wetland currently supports wintering population of White-naped Crane (*Grusvipio*), species listed as vulnerable and Tundra Bean Goose (*Ansercygnoides*), spring-autumn migration population of Swan Goose (*Ansercygnoid*), species listed as vulnerable and a breeding population of Black-faced Spoonbill (*Plataleaminor*), species listed as endangered in summer.

**New information:**

We provide data that the Janghang Wetland is a significant area for migration and breeding for waterbirds; and that Han River estuary is also internationally important for waterbirds during the migratory bird season. We observed 14 orders, 42 families and 132 species. The surveys also observed the critically-endangered Black-faced Spoonbill (*Plataleaminor*), Swan Goose (*Ansercygnoides*), White-naped Crane (*Grusvipio*), Whooper Swan (*Cygnuscygnus*) and Peregrine Falcon (*Falcoperegrinus*). We also observed the Black-faced Spoonbill, Great Egret, Little Egret, Great Cormorant, Eastern Spot-billed Duck, Pheasant and Brown-eared Bulbul at the sensor camera point and White-naped Crane, Hooded Crane, Bean Goose, White-fronted Goose, Snow Goose, Swan Goose, Great Cormorant and Eastern Spot-billed Duck at the closed-circuit television camera point from the camera-trap surveys. Based on the species recorded, the survey area is of clear importance for biodiversity conservation.

## Introduction

The Janghang Wetland is a site of international importance located in Han River estuary. It is an estuarine wetland teeming with a stretch of *Salixkoreensis* (Korean willows), rarely seen in other brackish water zones of Korea. The *Salixkoreensis* community has not only a symbiotic relationship with benthos, including *Chiromantesdehaani*, *Sesarmopsintermedius* and *Ilyoplaxdeschampsi*, which are indicator species of a blackish water zone, but also plays a role in regulating temperature of the urban area, decreasing carbon and protecting the margins of the river. It serves as an important stopover site for more than 30,000 birds each year, providing habitat and food for winter visitors, such as Black-faced Spoonbill (*Plataleaminor*), Hooded crane (*Grusmonachal*), White-naped Crane (*Grusvipio*) and Bean goose (*Anserfabalis*). This site was established as a National Wetland Protected Area on 17 April 2006, designated as East Asian-Australasian Flyway site on 10 May 2019 (EAAFP 2019) and Ramsar site on 20 May 2021 (Ramsar Convention Secretariat 2021).

## General description

### Purpose

MoE has surveyed the annual Winterbird Census since 1999, as part of the Asia Waterbird Census (MoE 1999, MoE and National Institute of Environmental Research 2000, MoE 2001, MoE and National Institute of Environmental Research 2002, MoE and National Institute of Environmental Research 2003, MoE and National Institute of Environmental Research 2004, MoE and National Institute of Environmental Research 2005, MoE and National Institute of Environmental Research 2006, MoE and National Institute of Environmental Research 2007, MoE and National Institute Biological Resources 2008, National Institute Biological Resources 2010, National Institute Biological Resources 2012, MoE and National Institute Biological Resources 2013). However, the current area of the Asia Waterbird Census does not include the westernmost part of the Han River estuary area, including Siman-ri Wetland in Gimpo-si (city) and Janghang wetland in Goyang-si (National Institute Biological Resource 2020, National Institute of Biological Resources 2022). In both areas, there are, at this time, thousands of geese, plus numerous other waterbirds. With this background, this work aimed to conduct an inventory of the waterbirds in the inner border area of Goyang in ROK, focused on the Janghang Wetland.

### Additional information

The survey result is provided in the supplementary material (Suppl. material 1).

## Project description

### Title

Ecological Survey in Janghang Wetland

### Personnel

The survey was led by a citizen-science group, including four experts who have experience over 10 years, 10 assistants who have experience over 5 years, one officer within local government and two ornithologists for identification and data preparation at research institutes. To ensure accuracy for the bird identification, we recorded photos and did peer-reviews. The survey was conducted over 11 months between April 2020 and March 2021 by ECO Korea, a citizen-science organisation. We gathered data during forty-four surveys.

### Study area description

**Figure 1.**
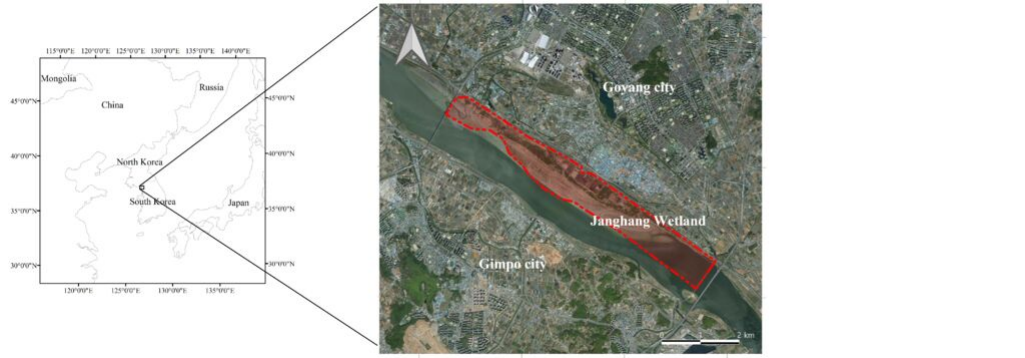
The survey area, Janghang Wetland (Sourced by authors' compilation, based on the map of Janghang Wetland by Goynag City).

The study area is part of Han River Estuary Wetland Protected Area, designated on 17 April 2006 by the Ministry of Environment, ROK. The total area is 60,668 km^2^ (Fig. 1). In addition, the study area is a civilian-controlled area in the DMZ, where access to the public is restricted due to the threat of land mines and the Military Installation Protected Area.

### Funding

The project is supported by the Basic Science Research Program through the National Research Foundation of Korea, funded by the Ministry of Education (NRF-2021R1A6A1A10045235) and Han River Basin Environmental Office.

## Sampling methods

### Study extent

This study covered Janhang Wetland and the surrounding area along the Jayu-Ro (road).

### Sampling description

**Figure 2.**
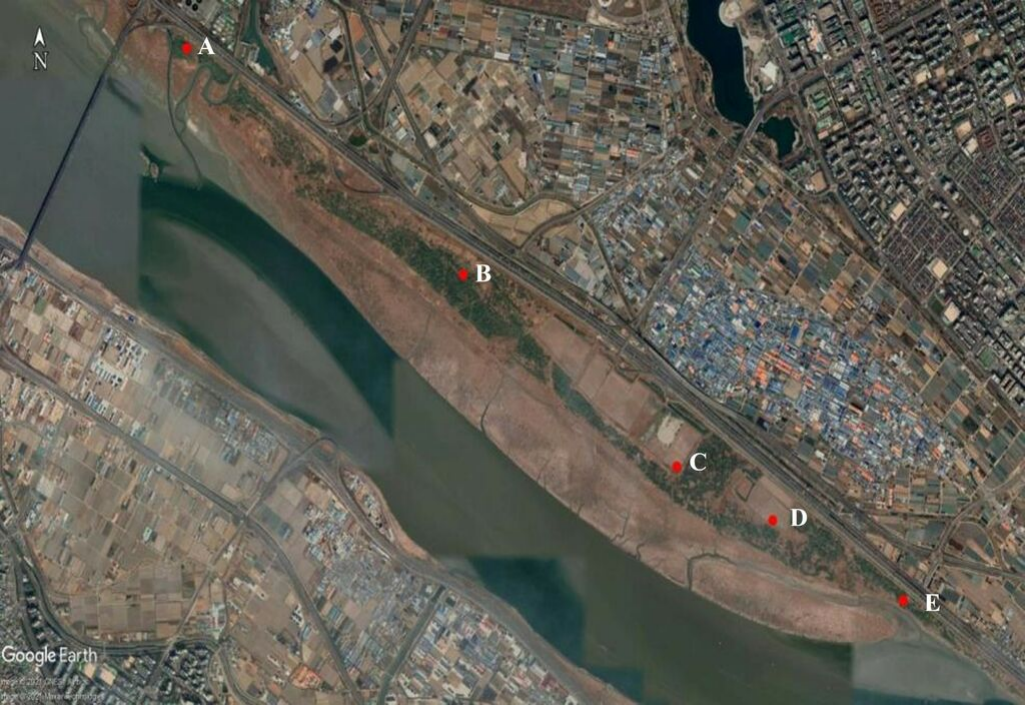
The survey points (A-E), sensor camera point (B) and closed-circuit television camera point (C) (Sourced by authors' compilation, based on Google Earth).

We focused counting on bird species found in open wetland habitats, as defined by the Ramsar Convention (e.g. along streams and waterways; the river edge). We covered five points with specific and secured areas (Fig. 2) and stayed for a particular time to survey with binoculars (8×32, Swarovski) and telescope (20~60×, Swarovski). We also did camera-trap surveys to record the cryptic species at the tidal channel, the inner area of Janghang Wetland, such as with a sensor camera (HP2X Hyperfire 2 Covert IR Camera) and a closed-circuit television camera at the submerged sleeping ground of the winter birds. During the surveys, we counted every individual bird, based on point surveys and line surveys that we either heard or saw from a slowly moving vehicle (average < 20 km/h) and walking in Janhang Wetland. We also did video analysis using camera trapping for the bird identification.

## Geographic coverage

### Description

The survey area covered the Goyang City of ROK, N37º39’22.36"~ 37º36’43.90" and E126º47’46.34"~126º43’3.43".

## Taxonomic coverage

### Description

This dataset (Suppl. material 1) provides the distribution information for the 132 species of birds recorded in Janghang Wetland following the IOC World Bird List version 13.1 (Gill et al. 2023).

### Taxa included

**Table:** 

Rank	Scientific Name	Common Name
kingdom	Animalia	Animals
class	Aves	Birds
order	Accipitriformes	
order	Anseriformes	
order	Bucerotiformes	
order	Charadriformes	
order	Ciconiiformes	
order	Columbiformes	
order	Cuculiformes	
order	Falconiformes	
order	Galliformes	
order	Gruiformes	
order	Passeriformes	
order	Pelecaniformes	
order	Piciformes	
order	Podicipediformes	
family	Accipitridae	
family	Acrocephalidae	
family	Aegithalidae	
family	Alaudidae	
family	Alcedinidae	
family	Anatidae	
family	Cettidae	
family	Charadriidae	
family	Ciconiidae	
family	Columbidae	
family	Coraciidae	
family	Corvidae	
family	Emberizidae	
family	Falconidae	
family	Fringillidae	
family	Gruidae	
family	Hirundinidae	
family	Laniidae	
family	Laridae	
family	Motacillidae	
family	Muscicapidae	
family	Oriolidae	
family	Pandionidae	
family	Paridae	
family	Passeridae	
family	Phalacrocoracidae	
family	Phasianidae	
family	Phylloscipidae	
family	Picidae	
family	Podicipedidae	
family	Pycnonotidae	
family	Rallidae	
family	Recurvirostridae	
order	Reguliidae	
family	Remizidae	
family	Scolopacidae	
family	Sturnidae	
family	Sylviidae	
family	Threskiornithidae	
family	Troglodytidae	
family	Turdidae	
family	Upupidae	

## Temporal coverage

### Notes

6 April 2020 to 29 March 2021

## Usage licence

### Usage licence

Creative Commons Public Domain Waiver (CC-Zero)

## Data resources

### Data package title

Janghang Wetland Avian Survey Data

### Number of data sets

1

### Data set 1.

#### Data set name

Avian Survey result

#### Data format

https://ipt.pensoft.net/resource?r=janghang

#### Data format version

Darwin Core Archive

#### Description

The dataset (Suppl. material 1) is the observation birds list in Janghang Wetland, Goyang City, ROK, 43x observations from April 2020 to March 2021. The taxonomy and nomenclature follow the IOC World Bird List (v. 13.1) (Gill et al. 2023).

**Data set 1.** 

Column label	Column description
basisOfRecord	The specific nature of the data record.
occurrenceID	An identifier for the Occurrence (as opposed to a particular digital record of the occurrence).
recordedBy	A list of names of people, groups or organisations responsible for recording the original Occurrence.
eventDate	The date-time or interval during which an Event occurred.
eventTime	The time or interval during which an Event occurred.
eventRemarks	Weather conditions.
continent	The name of the continent in which the Location occurs.
countryCode	The standard code for the country in which the Location occurs.
country	The name of the country or major administrative unit in which the Location occurs.
locality	The specific description of the place.
locationRemarks	Comments or notes about the Location.
decimalLatitude	The geographic latitude (in decimal degrees, using the spatial reference system given in geodeticDatum) of the geographic centre of a Location.
decimalLongitude	The geographic longitude (in decimal degrees, using the spatial reference system given in geodeticDatum) of the geographic centre of a Location.
geodeticDatum	The ellipsoid, geodetic datum or spatial reference system (SRS) upon which the geographic coordinates given in decimalLatitude and decimalLongitude are based.
coordinateUncertaintyInMetres	The horizontal distance (in metres) from the given decimalLatitude and decimalLongitude describing the smallest circle containing the whole of the Location. Leave the value empty if the uncertainty is unknown, cannot be estimated or is not applicable (because there are no coordinates). Zero is not a valid value for this term.
class	Class name.
order	Order.
family	Family.
scientificName	An identifier for the nomenclatural (not taxonomic) details of a scientific name.
vernacularName	A common or vernacular name.
individualCount	The number of individuals represented present.
identificationReferences	List of references (publications) used in the Identification.

## Supplementary Material

7DC3E1E4-8646-5C83-8B4C-BB7EDEDEBB7E 10.3897/BDJ.11.e105580.suppl1 7997176 Supplementary material 1 Janghang Wetland Avian Survey Data Data type Occurrences File: oo_842721.csv https://binary.pensoft.net/file/842721 Hyun-Ah Choi, Eunjeong Lee, Eunjeong Kim, Insook Jung, Donguk Han

## References

[B9712777] EAAFP New Flyway Network Sites in the Republic of Korea – Janghang Wetland and Incheon Songdo Tidal Flat. https://www.eaaflyway.net/new-flyway-network-rok/..

[B9714774] Gill F, Donsker D, Rasmussen P (2023). IOC World Bird List (v13.1)..

[B9713370] MoE (1999). Winter Waterbird Census of Korea.

[B9713378] MoE, Research National Institute of Environmental (2000). Winter Waterbird Census of Korea.

[B9713386] MoE (2001). Winter Waterbird Census of Korea.

[B9713394] MoE, Research National Institute of Environmental (2002). Winter Waterbird Census of Korea.

[B9713402] MoE, Research National Institute of Environmental (2003). 2003 Winter Waterbird Census of Korea.

[B9713410] MoE, Research National Institute of Environmental (2004). 1999-2004 Comprehensive Report of Winter Waterbird Census of Korea.

[B9713418] MoE, Research National Institute of Environmental (2005). 2005 Winter Waterbird Census of Korea.

[B9713426] MoE, Research National Institute of Environmental (2006). 2006 Winter Waterbird Census of Korea.

[B9713442] MoE, Research National Institute of Environmental (2007). 2007 Winter Waterbird Census of Korea.

[B9713458] MoE, Resources National Institute Biological (2008). 2008 Winter Waterbird Census of Korea.

[B9713490] MoE, Resources National Institute Biological (2013). 2013 Winter Waterbird Census of Korea.

[B9713517] Resource National Institute Biological (2020). 2019-2020 Winter Waterbird Census of Korea.

[B9713466] Resources National Institute Biological (2010). 2010 Winter Waterbird Census of Korea.

[B9713474] Resources National Institute Biological (2012). 2012 Winter Waterbird Census of Korea.

[B9712765] Resources National Institute of Biological (2022). 2021-2022 Winter Waterbird Census of Korea.

[B9712785] Secretariat Ramsar Convention Republic of Korea adds Janghang Wetland to the List. https://ramsar.org/news/republic-of-korea-adds-janghang-wetland-to-the-list.

